# A Review of over Fourteen Thousand Surgical Anæthesias

**Published:** 1907-03

**Authors:** 


### A REVIEW OF OVER FOURTEEN THOUSAND SURGICAL ANAESTHESIAS.

---

Alice McGraw states that at St. Mary's Hospital their preference of anæsthetics has always been ether. They have tried all of the combinations, but have always come back to the "drop method" of giving ether. They use a four-ounce ether can and fit an ordinary cork with a groove on either side into its mouth, fill one groove with absorbent cotton and let it extend out of the can about one inch, regulating the drop by the manner in which the point is clipped. She uses two cans, one with a large dropper until the patient is fully anæsthetized, and then one with a smaller dropper. This inhaler used is the improved Esmarch, with two thicknesses of stockinet (frame boiled and stockinet changed after each patient). She talks to the patient all the while to gain his confidence, but she does not allow him to talk or count or breathe deep. She holds that suggestion is a great aid in producing a comfortable narcosis. The patient is placed in position on the table before being anæsthetized and his preparation is begun at the same time as the anæsthetic. She relies upon his respiration, color, and relaxation of the jaw as to the stage of the anæsthesia. Respiration is of very much more importance than the pulse and only the inexperienced take the pulse and touch the conjunctive when giving ether. Gall bladder cases are very difficult on account of the nearness to the diaphragm; stomach cases are prone to be followed by pneumonias, and very little ether should be used. She thinks nurses make better anæsthetists than physicians because they have no ambition to become surgeons.—Surg. Gynecol. & Obstetrics.

